# Part I: Development and Implementation of the Ten, Five, Three (TFT) Model for Resistance Training

**DOI:** 10.3390/muscles4020014

**Published:** 2025-05-19

**Authors:** Quincy R. Johnson

**Affiliations:** Jayhawk Athletic Performance Laboratory—Wu Tsai Human Performance Alliance, Department of Health, Sport and Exercise Sciences, University of Kansas, Lawrence, KS 66045, USA; quincy.johnson@ku.edu; Tel.: +1-785-864-1944

**Keywords:** strength and conditioning, systems, frameworks, LTAD, performance, injury, program design, periodization

## Abstract

The strength and conditioning literature examining neuromuscular physiology, bioenergetics, neuroendocrine factors, nutrition and metabolic factors, and the use of ergogenic aids, as well as physical and physiological responses and adaptations, have clearly identified the benefits of participating in regular resistance training programs for athletic populations, especially as it relates to improving muscular strength. Beyond evidence-based research, models for resistance training program implementation are of considerable value for optimizing athletic performance. In fact, several have been provided that address general to specific characteristics of athleticism (i.e., strength endurance, muscular strength, and muscular power) through resistance training over the decades. For instance, a published model known as the strength–endurance continuum that enhances dynamic correspondence (i.e., training specificity) in athletic populations by developing structural, metabolic, and neural capacities across a high-load, low-repetition and low-load, high-repetition range. Further models have been developed to enhance performance approaches (i.e., optimum performance training model) and outcomes (i.e., performance pyramid), even within specific populations, such as youth (i.e., youth physical development model). The ten, five, three, or 10-5-3 (TFT) model for strength and conditioning professionals synthesizes currently available information and provides a framework for the effective implementation of resistance training approaches to suit the needs of athletes at each stage of development. The model includes three key components to consider when designing strength and conditioning programs, denoted by the acronym TFT (ten, five, three). Over recent years, the model has gained much support from teams, coaches, and athletes, mainly due to the ability to streamline common knowledge within the field into an efficient and effective resistance training system. Furthermore, this model is distinctly unique from others as it prioritizes the development of strength–endurance, muscular strength, and muscular power concurrently. This paper explains the model itself and begins to provide recommendations for those interested in implementing TFT-based approaches, including a summary of points as a brief take-home guide to implementing TFT interventions. It is the author’s hope that this paper encourages other performance professionals to share their models to appreciate human ingenuity and advance our understanding of individualized approaches and systems towards the physical development of the modern-day athlete.

## 1. Introduction

Muscular strength is a key contributor to athletic performance, with an array of literature supporting its importance for the modern-day athlete, regardless of sport or level of competition [1,2,3,4,5,6,7,8,9]. Based on the current understanding about the benefits of resistance training, athletic organizations across competition levels implement it in some form to mitigate injury risk and enhance athletic performance. In recognition of this apparent consensus that muscular strength is an important characteristic within athletic populations, there have been many models developed and reported in the strength training literature. These models highlight resistance training approaches that can be used in consideration of specific aims (i.e., muscular strength development, muscular power development, etc.), common constraints (i.e., limited time, resources, personnel, etc.), and population-specific needs (i.e., amateur to professional) [9,10,11,12,13,14,15,16,17,18,19,20]. However, challenges exist for applied performance professionals, teams, coaches, and athletes regarding synthesizing the available information related to best approaches for resistance training and adapting it to suit their specific needs and goals. To address these challenges, an adaptable model has been developed that can be integrated into most settings, for most athletes, and does not require coaches to have advanced knowledge besides what is required to enter and succeed within the field of strength and conditioning.

Realizing the need for a model based on solid theoretical and empirical foundations to help guide the use of resistance training approaches, the ten, five, three, or 10-5-3 (TFT) resistance training approach was devised, which, after successful implementation, has been formalized into its current model form. TFT is an acronym representing a three-fold approach to be followed when developing and implementing resistance training programming. To be clearly stated, this model’s primary focus is to enhance an individual’s physical preparedness, robustness and resilience, muscular strength, and muscular power that can be translated to indices of athletic performance. The TFT model is based on evidence-based findings and models from sport science [21], strength and conditioning [1,2,3,8,9,22,23,24,25,26,27,28,29,30,31,32,33], neuromuscular physiology [34,35,36,37,38,39,40], bioenergetics [41,42,43,44,45,46], neuroendocrine factors [47,48,49,50,51,52,53,54,55,56], nutrition and metabolic factors [57,58,59,60,61,62,63,64,65,66], the use of ergogenic aids [67,68,69,70,71,72,73,74,75,76], physical and physiological responses and adaptations to exercise [77,78,79,80,81,82,83,84,85,86,87,88,89], sport psychology [90,91,92,93,94], and ecological dynamics theory [95,96,97,98,99,100]. Ultimately, the TFT model aims to provide practitioners with a set of practical guidelines to support their strength and conditioning programming.

Perhaps the most fundamental difference between the TFT model and the more traditional resistance training models proposed is that resistance training has often been thought of as a rigid and focused effort towards maximizing muscular strength. However, the TFT model posits that if a clear goal or benchmark is determined, the fluid development of physical fitness, muscular strength, and muscular power simultaneously is possible. Furthermore, this approach towards resistance training program design and implementation may be advantageous for the development of the modern-day athlete based on the increasing demands of competitive athletics (e.g., increased competitions, early sport specialization, increased access to strength and conditioning programming). Time available to train is a major constraint in most strength and conditioning programs. This constraint has created a gap between program goals and identifying effective and efficient methods for attaining them. The TFT model aims to bridge this gap. For example, resistance training programs for basketball athletes that regularly include exercises that address strength endurance, muscular strength, and muscular power simultaneously will be a closer representation of what those athletes will experience at different times throughout training and competition (e.g., rebound, pass, transition, catch, layup) compared to a singular focus on muscular strength. Another example is that which has been provided by the 2015 National Football League’s (NFL) Strength and Conditioning Coach of the Year recipient, Joe Kenn, founder of the “Tier System” [5]. Throughout his career and within his published book, “The Coach’s Strength Training Playbook: Featuring the Tier System”, Coach Kenn specifically highlights the demands of sport at the professional level and thus the specific needs of elite athletes who are preparing to regularly compete, whether in training or competition.

**Figure 1:**
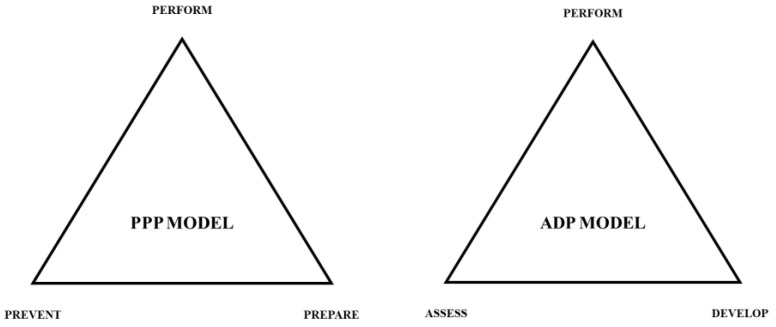
The three components of the overarching PPP and ADP models.

**Figure 2:**
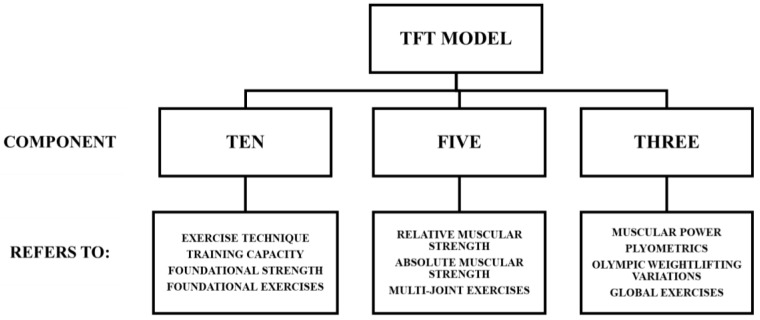
The three components of the TFT model supported by prior findings [2].

**Figure 3:**
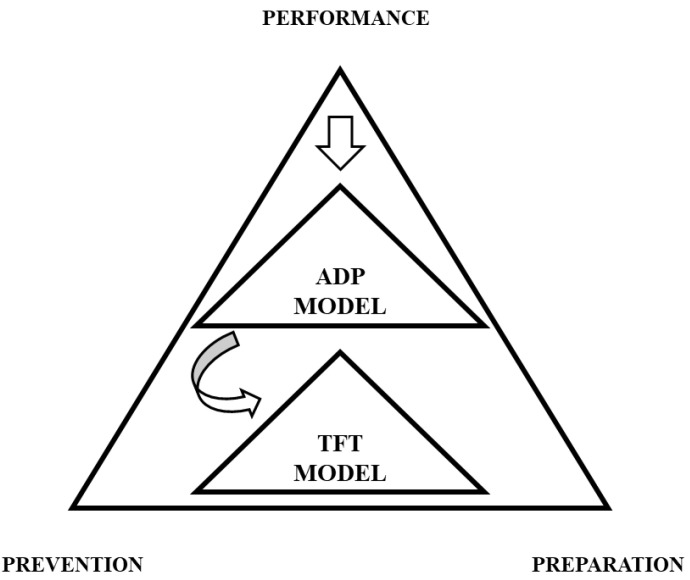
An example of how the ADP and TFT models fit within the PPP model.

**Figure 4:**
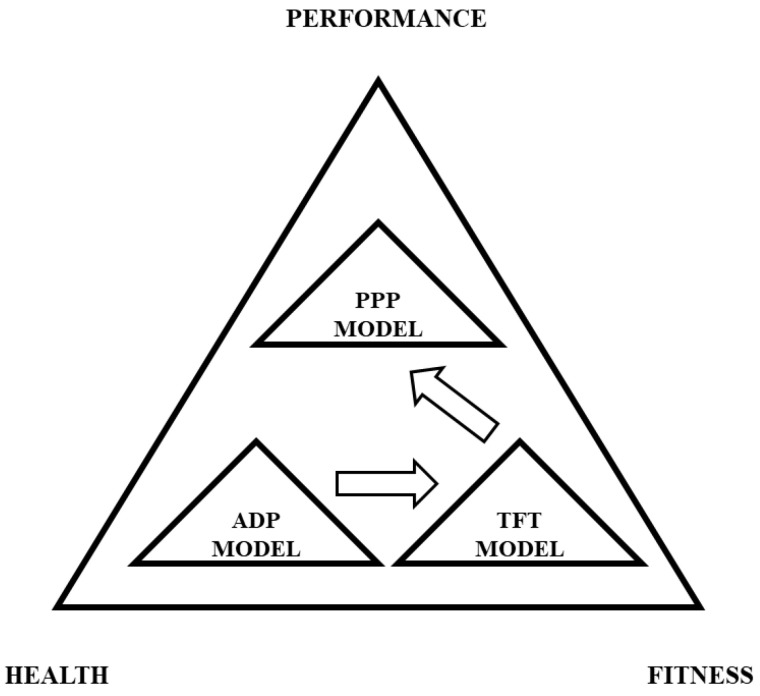
An example of how the ADP, TFT, and PPP models fit within the TTCS model.

It should be noted that the TFT model is but one model directly related to another that is encapsulated by one broader model that can be used to guide resistance training approaches for athletic populations. Figure 1 illustrates the broader prevent, prepare, performance (PPP) model, which has synthesized evidence-based findings from strength and conditioning as well as the sport science literature in order to consider each primary component of strength and conditioning programming to support athletic performance [1,2,3,4,5,21,22,23,24,25,26,27,28,29,30,31,32,33]. Figure 2 illustrates the assess, develop, perform (ADP) model, which fits within the broader PPP model, which has also synthesized evidence-based findings from the literature in order to streamline the process of implementing testing and assessment, athlete monitoring, resistance training, and sport science approaches. Figure 3 illustrates the TFT model, which can be used to assist with the program design and implementation of resistance training approaches. Finally, Figure 4 illustrates the triple triangle complex system model (TTCS), which encapsulates each of the three models utilized to enhance physical and physiological development of the athlete. The primary objective of this manuscript is to introduce a conceptual resistance training model that effectively and efficiently enhances strength–endurance, muscular strength, and muscular power.

## 2. Materials and Methods

### 2.1. The Three Components of the PPP Model

#### 2.1.1. Prevent

The component “Prevent” refers to the importance of utilizing resistance training methods to contribute to the prevention or mitigation of injury risk within athletic populations [101,102,103,104,105,106]. Ample evidence suggests that in addition to increasing muscular strength and hypertrophy (i.e., contractile tissue), resistance training promotes increases in the strength of ligaments, tendons, joint cartilage, connective tissue sheaths within muscle, and bone mineral density across an array of populations [101,102,103,104,105,106]. This preventative component to resistance training program design and implementation is best utilized with the inclusion of not only corrective or rehabilitative exercises but also exercises that develop muscular strength as a protective measure during sport-related activities.

#### 2.1.2. Prepare

The component “Prepare” refers to the importance of adequate physical preparation within athletic populations to withstand the demands of training and competition with an ultimate aim of supporting optimal performance. The foundational strength and conditioning literature has highlighted the importance of physical preparation dating back as far as the ancient military training of the Chinese, Egyptians, Greeks, and Romans and transcending time to the more modern literature and approaches adapted for the modern-day sportsman and sportswoman [107,108,109,110,111,112]. Generally, the literature suggests that adequate physical preparation follows a sequence of general to specific approaches which aim to enhance exercise technique and energy system development first and then muscular strength and muscular power over a well-measured period of time [107,108,109,110,111,112]. This preparation component for resistance training program design and implementation can be used alongside the preventative component to achieve the primary component, which is performance.

#### 2.1.3. Perform

The component “Perform” refers to the importance of utilizing resistance training models, modes, and methods to support the primary objective of most sporting organizations and teams: optimal athletic performance [89]. However, the author posits that this component can only be achieved consistently with a thorough understanding of sport science [21], strength and conditioning [1,2,3,8,9,22,23,24,25,26,27,28,29,30,31,32,33], neuromuscular physiology [34,35,36,37,38,39,40], bioenergetics [41,42,43,44,45,46], neuroendocrine factors [47,48,49,50,51,52,53,54,55,56], nutrition and metabolic factors [57,58,59,60,61,62,63,64,65,66], the use of ergogenic aids [67,68,69,70,71,72,73,74,75,76], physical and physiological responses and adaptations to exercise [77,78,79,80,81,82,83,84,85,86,87,88,89], sport psychology [90,91,92,93,94], and ecological dynamics theory [95,96,97,98,99,100], as well as how each of the foundational level components (i.e., prevent and prepare) interact with one another and can be adapted to make progress towards this chief objective.

### 2.2. The Three Components of the ADP Model

#### 2.2.1. Assess

The component “Assess” refers to the importance of assessing performance and fatigue within athletic populations to not only understand an athlete’s strengths, weaknesses, and responses to training programs but also to adjust approaches if need be to ensure positive adaptation occurs. Prior evidence across the strength and conditioning as well as sport science literature has suggested the importance, validity, reliability, and many benefits of assessing athletic populations to support the aim of achieving optimal athletic performance [113,114,115,116,117,118,119,120]. This assessment component of resistance training program design and implementation plays a critical role in the identification or creation of developmental approaches to be implemented that can enhance physical and physiological characteristics that contribute to optimal athletic performance. For instance, this component can be integrated into regular physical fitness testing that can occur during pre-, mid-, and post-season timepoints and can range from non-fatiguing to fatiguing. However, new developments in technology and its seamless integration into athletic departments have allowed for regular non-invasive assessments to be integrated into resistance training sessions of the modern-day American college football athlete following their warmup. For example, an assessment of lower-body neuromuscular performance and fatigue via the countermovement vertical jump on force plates can provide practitioners with force–time characteristic-related data (i.e., braking force, power, and velocity, propulsive force, power, velocity, reactive strength index, etc.) that can be used to guide program design, practice design, exercise selection, or on a broader scale periodization approaches.

#### 2.2.2. Develop

The component “Develop” refers to the importance of (1) developing specific physical and physiological characteristics within athletic populations to support optimal performance and (2) the resistance training means, methods, and modes implemented to achieve this goal [90]. Furthermore, this component aligns well with the “prepare” component included within the PPP model but can be viewed as a more detailed approach towards resistance training program design and implementation. While the strength and conditioning literature provides vast developmental approaches for athletes at different competitive levels, the identification, prioritization, and streamlining of this component is based on both experience and evidence, as well as the consideration of constraints specific to each environment that are necessary within the athletic environment [8,9,10,11,95,96,97,98,99,100]. Beyond periodization and program design, this component should be carefully considered, especially as it relates to the development of specific characteristics, such as muscular strength within athletic populations and the systematic approach for how they should be developed. Strength and conditioning professionals should consider not only the foundational elements of a comprehensive resistance training program (i.e., accounting for volumes, loads, intensities, training frequency, etc.) but also the more in-depth elements, such as the training culture and philosophy towards athlete development that materializes into the environment created during the training process in conjunction with the exercises selected and technologies utilized (e.g., velocity based training), to, of course, support optimal athletic performance [121,122]. Within the resistance training setting for collegiate American football athletes and many other sports, systems of development can contribute to the immediate and longer-term development of general and specific physical qualities, as well as ensure that consistent approaches are being implemented across the coaching staff. An added benefit to a system of physical development is the assessment of its effectiveness.

#### 2.2.3. Perform

The component “Perform” ultimately aligns with performance-related information reported within the PPP model but should also be adapted to evaluate and support optimal athletic performance within sport-specific training and competition environments. Further, this component can be specifically focused towards either standard performance statistics from competition or the subsequent data (i.e., biometrics, total distances covered, physical workload, etc.) from implemented microtechnology (i.e., global or local positioning systems, heart rate monitors, accelerometers, etc.) [21,121,122,123,124,125,126,127,128,129,130]. For the sport performance practitioner, a model such as this cannot only enhance the understanding of how each component contributes to the next but also how each can be aligned and adapted to support this higher-order objective as well as how information from this objective can be regressed to fit within developmental systems and guide assessment methods.

### 2.3. The Three Components of the TFT Model

#### 2.3.1. Ten

The component “Ten” refers to the importance of the ten-repetition range for developing exercise technique, strength endurance, training capacity, and foundational muscular strength through the prioritization of foundational exercise implementation [41,131,132,133,134,135,136,137,138,139,140,141,142,143,144]. The immense value of muscular endurance, particularly that of strength endurance, has been well reported in prior research. In particular, the Stone et al., 2006 publication clearly establishes the benefit of high-volume training approaches within the athletic population based on both practical experience and evidence-based scientific approaches [144]. To that end, muscular endurance is a foundational element that contributes to developing muscular strength, muscular power, and overall athleticism. Furthermore, the development of muscular endurance at lower intensities is critical for improving exercise technique, body composition, metabolic efficiency, and preparing the body’s tissues for higher and more intense loads [144]. Prior evidence suggests that intensities lower than 67% of 1-repetition maximum are adequate for developing muscular endurance [4]. What first began as a foundational element to the TFT model to ensure that athletes were developing adequate exercise technique, physical fitness, and foundational strength has come to play a critical role in the ability of athletes to sustain physical activity for longer periods of time at high intensities, low to moderate loads, and higher training densities, such as that expressed by the three MMA professional level athletes who attained championship-caliber performances by utilizing this system of training as well as several other athletes across sport [139,140,141,142,143]. The importance of performing repetitions within the 10-repetition range cannot be overstated.

Reported benefits of resistance exercise within the 10-repetition range include [2,132,133,134,135,136,137,138,144] the following:

- Improvements in body composition;
- Improved metabolic alterations, responses to stressors, and work capacity;
- Improvements in strength–endurance and power–endurance;
- Substantial increases in testosterone and growth hormone concentrations postexercise;
- Increased resting testosterone–cortisol ratio;
- Adequate development of physiological foundation for further, more specific resistance training.

The following is a brief list of foundational exercises as suggested in the National Strength and Conditioning Association’s Basics of Strength and Conditioning Manual [4]:

- Squat;
- Step;
- Hinge;
- Lunge;
- Push;
- Pull;
- Carry.

#### 2.3.2. Five

The component “Five” refers to the importance of the 5-repetition range for developing absolute and relative—as well as general and specific—muscular strength to withstand the physical and physiological stress of training and competitive demands, as well as to express optimal ground reactive forces. The well-established strength training literature has established the five-repetition range of multi-joint compound exercises as sufficient for developing muscular strength within most athletic populations [2,131,132,133,134,135,136,137,144]. Furthermore, this literature has also highlighted how the ability to produce force maximally and at higher absolute values significantly correlates and contributes to significantly higher muscular power and sprinting speed capabilities. Prior evidence suggests that intensities between 75 and 85% of 1-repetition maximum are adequate for developing muscular strength within the 5-repetition range [4]. Within the TFT model, exercises are programmed in trios. That is, there are typically three exercises to be performed within the 10-repetition range, three exercises within the 5-repetition range, and three exercises within the 3-repetition range. With that said, this model often utilizes one foundational muscular strength exercise alongside two variations of other foundational strength exercises. For instance, a boxer primarily utilizes their upper extremities to complete sporting actions, but the practitioner knows that force begins at the ground. When designing an upper-body resistance training program using the TFT model, the practitioner would program in a barbell back squat, alongside a goblet squat, and pullups. By approaching muscular strength development in this fashion, not only are the necessary muscles developed but training also becomes more efficient, and the overall physical development of the athlete is likely more robust.

The following is a brief list of multi-joint exercises to develop muscular strength as suggested in the National Strength and Conditioning Association’s Basics of Strength and Conditioning Manual [4]:

- Barbell back squat;
- Barbell front squat;
- Barbell bench press;
- Barbell incline bench press;
- Barbell overhead press;
- Barbell deadlift;
- Trap bar deadlift.

#### 2.3.3. Three

The component “Three” refers to the importance of the 3-repetition range for developing muscular power. This can be achieved by focusing on transferring muscular strength capabilities to the velocity and time-dependent characteristics of training and competitive demands. Prior evidence suggests that intensities between 75 and 85% or lower than 30% of 1-repetition maximum are adequate for developing muscular power [4]. In alignment with prior findings, exercises that are most adequate for developing this type of physical characteristic are those that are explosive, ballistic, plyometric, or include Olympic weightlifting variations [131,132,133,134,135,136,137,144]. Furthermore, and beyond the repetition range, is the method of implementing training to ensure that athletes are properly recovered between sets and can train at maximal intensities. The TFT model has leveraged existing knowledge provided by Kenn, Stone et al., Tufano et al., and Haff et al. in regard to the clustered nature of training for enhanced training intent, training intensity, and transfer to sporting performance, and this is a critical element to the TFT model that will be explained in subsequent publications [5,22,23,28,144].

The following is a brief list of exercises to develop muscular power as suggested in the National Strength and Conditioning Association’s Basics of Strength and Conditioning Manual [4]:

- Landing;
- Jumping;
- Throwing;
- Clean variations;
- Jerk variations;
- Snatch variations.

### 2.4. Using the TFT Model to Guide Practice

**Figure 5:**
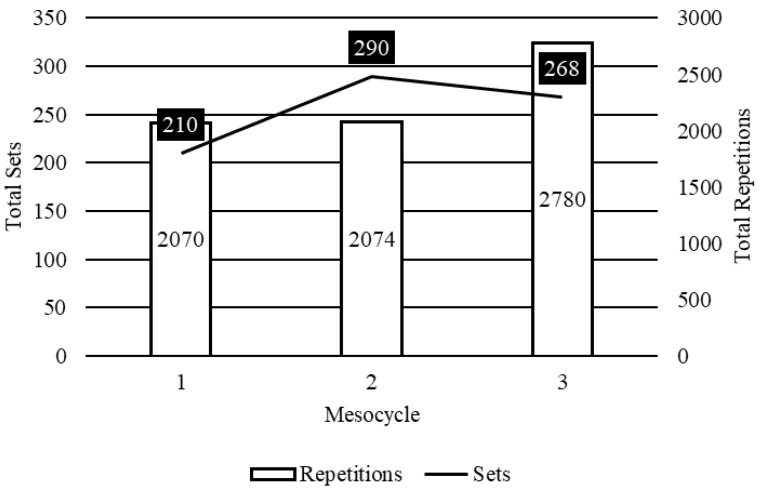
Sample set and repetition characteristics for youth athlete populations trained utilizing the TFT model. When compared to the adult model, it is noticeable that training volumes are higher throughout most cycles but dispersed over more sets for youth. This highlights another benefit related to the potential for longer-term development of athletes within an organized system of training that includes the TFT model.

**Figure 6:**
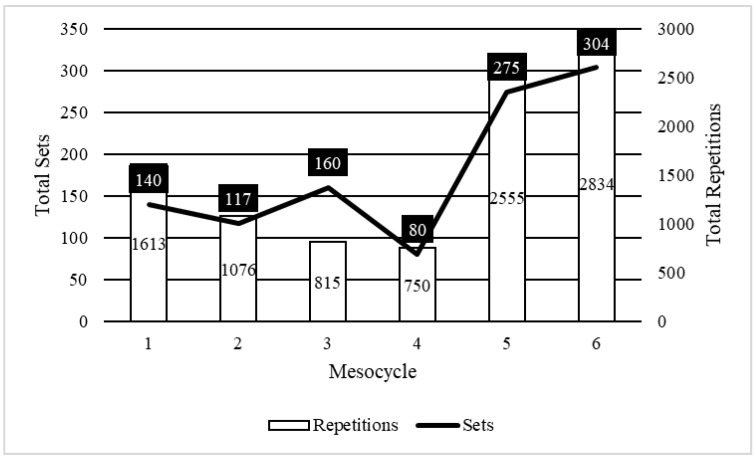
Sample set and repetition characteristics for professional athlete populations who attained championship outcomes by utilizing the TFT model.

The TFT model is best used in practice by implementing each component within a single session in a circuit-like fashion. For instance, a specific portion of the training session should be dedicated towards developing training technique and capacity by utilizing the ten-repetition range before proceeding to a specific portion of the training session dedicated towards developing muscular strength by utilizing the five-repetition range before concluding with a specific portion of the training session dedicated towards developing muscular power by utilizing the three-repetition range. Certainly, neuromuscular fatigue may be apparent during the resistance training session, but this would be the case in a competitive sport-specific environment as well, so this approach towards training may in fact better prepare athletes for those scenarios. In practice, the aforementioned approach would be used during the general physical preparatory period, and more specific approaches would be utilized closer to competition (Table 1). Furthermore, as the strength and conditioning program transitions between phases, the exercises within each phase should become more specific in order to adequately prepare the athlete for the demands of training and practice. For example, during the general physical preparation phase, a focus on bilateral exercises should be prioritized for most athletic populations, while during the specific physical preparation phase, a shift towards unilateral exercises or bilateral exercises performed at specific velocities should be prioritized. Additional information related to the TFT model is included in Table 2, Table 3 and Table 4. Further information related to pilot data and case study outcomes that illustrate the effectiveness of the model across competitive levels can be found in subsequent Table 5 and Table 6 and Figure 5 and Figure 6.

## 3. Conclusions

To conclude, athletic performance is underpinned by optimal physical, technical, tactical, and psychological performance. Resistance training is an effective method for developing athletes physically as they prepare for training and competition. Furthermore, evidence suggests that resistance training is a safe and effective method for enhancing physical and physiological qualities that specifically contribute to optimal athletic performance (i.e., muscular strength) without being sport specific. However, few models exist which aim to synthesize prior suggested evidence for application into practice, especially as it relates to benefiting the strength and conditioning practitioner who may face the challenge of limited time to train athletes in general or to prepare them for competition (e.g., transfer portal, academic calendar, competition calendar, etc.). Collectively, the TFT model addresses each of the three primary underlying components that contribute to the optimal preparation of athletes (i.e., strength endurance, muscular strength, and muscular power) and consolidates them to support a focused system and approach towards resistance training in a unique way.

It should be noted that the TFT model of resistance training program design and periodization is not without its limitations. The primary limitation is that it may be viewed as a general approach to improving athleticism via resistance training. Although the concepts that have been generalized are well accepted within the scientific community, an advanced understanding of exercise physiology as well as strength and conditioning was utilized to refine the current version of the model. Furthermore, a similar understanding may be needed to properly implement and modify this model for specific populations. The next limitation of the TFT model is that due to its general nature, athletes who may need more specific programming could experience fewer benefits. For instance, athletes who specifically need to develop muscular power may not necessarily benefit from one of the more general periodization models included within Table 1. However, for the skilled practitioner, adjusting training frequency, intensity, time, type, volume, and progression may address the specific needs of those athletes, and this model provides a systematic approach for doing so. Lastly, one of the final limitations of this model is that it requires practitioners to think “outside of the box”, that is, traditional program design and periodization approaches. Although generally effective, some traditional approaches are not very easily adaptable to the constraints of the modern-day athletic environment and needs of today’s athlete. Perhaps, it is quite possible to implement a system of resistance training, such as the TFT model, that concurrently addresses the safe and effective development of strength–endurance, muscular strength, and muscular power within athletic populations.

In the future, research related to validating this model of resistance training program design and periodization could explore several avenues. The first, and from an athletic performance outcome perspective, could include the retroactive analysis of resistance training characteristics such as volumes, intensities, and densities of successful athletes trained within this model (see Table 6 for preliminary analysis). To clarify, this is not to overshadow the importance of technical, tactical, and sport psychology mastery but to better understand a component that contributes to overall success. Next, a comparative study that aims to validate the TFT model versus other models of resistance training program design and periodization could be completed to highlight potential similarities and differences in physical and physiological adaptations. Lastly, longitudinal athlete monitoring should be explored to further understand long-term improvements in physical and physiological characteristics as well as athletic performance outcomes. Furthermore, subsequent models from the field should be published to further our current understanding of how prior evidence can be adapted to successfully prepare athletes for optimal performance. To conclude, the TFT model provides a unique contribution to the sports performance profession by synthesizing prior evidence into an effective and efficient system for athletic development via resistance training, specifically by utilizing evidence to identify repetition ranges (i.e., 10, 5, and 3) that correspond to ideal physical characteristics (i.e., strength–endurance, muscular strength, and muscular power) that can be adapted across sport and competition levels.

## Figures and Tables

**Table 1:** Example of the TFT model implemented during the general physical preparation (GPP), specific physical preparation (SPP), and competitive phases (CPs).

Emphasis	GPP	SPP	CP
1.	10-repetition range	5-repetition range	3-repetition range
2.	5-repetition range	3-repetition range	5-repetition range
3.	3-repetition range	10-repetition range	10-repetition range

**Table 2:** General progression of exercises for athletic performance used within the TFT model.

Quality	Regression 1	Base Exercise	Progression 1
Strength	Multi-joint/compound bodyweight movements	Multi-joint/compound loaded movements	Multi-joint/compound fast loaded movements
Power	Position and technique	Force-dominant Olympic weightlifting variations	Power-dominant Olympic weightlifting variations
Plyometrics	Landing mechanics	Single response/non-countermovement	Multi-response/countermovement

**Table 3:** Example of exercises used within each component of the TFT model.

Exercise Order	Ten	Five	Three
1.	Incline pushup	Barbell back squat	Jump landing technique
2.	Kettlebell goblet squat	Incline dumbbell chest press	Depth drop
3.	Inverted row	Dumbbell row	Box jump

**Table 4:** Exercise options adapted from “The Coach’s Strength Training Playbook: Featuring the Tier System” [5].

Lower Body	Upper Body	Total Body
Back squat	Bench press	Hang clean
Front squat	Dumbbell bench press	Split clean, hang
Box squat	Incline press	Clean pull, hang
Safety bar squat	Dumbbell incline press	Hang snatch
Bear squat	Modified grip bench press	Split snatch, hang
Leg press	Standing shoulder press	Snatch pull, hang
High step-up 16″	Nelder press	Jerk, split catch
Low step-up 6″	Bent over row	Jerk, power catch
Barbell lunge	Shrug	Push press
Romanian deadlift	Dumbbell lateral raise	Dumbbell clean, hang
Single-leg squat	Dip	Dumbbell snatch, hang
Leg curl	Chin-up	Dumbbell jerk
Leg extension	Triceps extension	
Calf raises	Biceps curl	

**Table 5:** Mesocyclic characteristics of the example TFT model implemented for amateur athletic populations.

Mesocycle	1	2	3
Sets	210	290	268
% Change		38.10%	−7.59%
Repetitions	2070	2074	2780
% Change		0.19%	34.04%
Repetitions/Set	9.86	7.15	10.37
% Change		−27.45%	45.04%
Sessions/Day	1	1	1
Days/Week	2	2	2
Intensity Cycle	3/1	3/1	3/1
Mesocycle	1	2	3
Sets	210	290	268

**Table 6:** Mesocyclic characteristics of the sample TFT model implemented for professional athletic populations who attained championship outcomes.

Mesocycle	1	2	3	4	5	6
Sets	140	117	160	80	275	304
% Change		−16.43%	36.75%	−50.00%	243.75%	10.55%
Repetitions	1613	1076	815	750	2555	2834
% Change		−33.29%	−24.26%	−7.98%	240.67%	10.92%
Repetitions/Set	11.52	9.20	5.09	9.38	9.29	9.32
% Change		−20.18%	−44.61%	84.05%	−0.90%	0.34%
Sessions/Day	1–2	1–2	1–2	1–2	1–2	1–2
Days/Week	3	3	3	3	3	3
Intensity Cycle	2–3/1	2–3/1	2–3/1	2–3/1	2–3/1	2–3/1

## Data Availability

The data presented in this study are available on request from the author.
